# Supplementary material for: Learning curve and surgical outcome of robotic assisted colorectal surgery with ERAS program
Source: Sci Rep. 2022 Nov 29;12:20566. doi: 10.1038/s41598-022-24665-w (PMC9709162; doi:10.1038/s41598-022-24665-w)
Supplement: Supplementary file 2 — Supplementary Information 2. [file 41598_2022_24665_MOESM2_ESM.zip › RAL Raw data-Ñ[▒K/123/OP_ñw╝╨░O▒Kñσ.pdf]

正式摘要(電子簽章：000000228930322)

W83 / W83

臺中榮民總醫院

Operation Note

[redacted]

- 手術日期：2021/11/02
- 手術醫師：VS 林俊余，CR 陳瑢夫，R 林易萱，R 蘇偉翔
- Anesthesia : GE
- Position : Supine
- Aseptic agents : Soap

Diagnosis

- Pre-operative diagnosis : s/p RRP 2007 with adhesion.
- Post-operative diagnosis : Ditto.s/p robotic adhesiolysis

Surgical Indication

- Bowel obstruction

Operation

- Robot Assisted Colectomy(L)

Finding

- severe adhesion of small intestine on Rt side abdomen cavity
  2 adhesion band was lysed by robotic arm.

Procedure

- 1. General endotracheal anesthesia was induced and then the patient was placed in supine position.

  2. Preoperative antibiotics were given. A Foley catheter and nasogastric tube were placed.
  3. The abdomen was prepped and draped in standard sterile fashion.
  4. A small vertical incision was made LUQ and a 8 mm trocar was inserted into the peritoneal cavity by open method. Peumoperitoneum was established with CO2 inflation.
  5. Laparoscopy was inserted and then other 8mm trocars were inserted over of abdomen. The operative findings were mentioned as above.
  6. Adhesiolysis was performed then and 3-0 Vicryls were applied for serosa repair if needed.
  7. the wounds was closed with interrupted 3-0 Nylon.
  8. The patient tolerated the procedure well and was brought to the POR in stable condition
- Estimated blood loss: minimum
- 器械數量: 正確
- 紗布數量: 正確

Image

- PAINT
  - abdomen

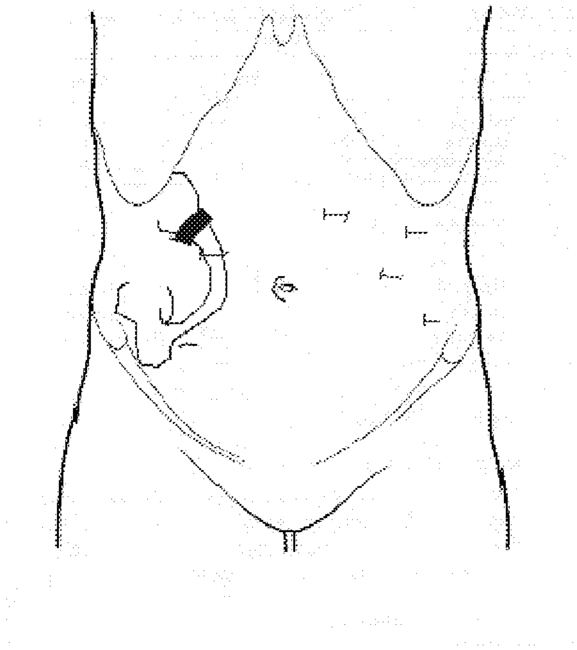

主治 2222H林俊余 / 醫師 2310K陳瑨夫

正式摘要(電子簽章：000000228930322)

W83 / W83

doctorRank:B
